# A self-adjuvanted nanoparticle based vaccine against infectious bronchitis virus

**DOI:** 10.1371/journal.pone.0203771

**Published:** 2018-09-14

**Authors:** Jianping Li, Zeinab H. Helal, Christopher P. Karch, Neha Mishra, Theodore Girshick, Antonio Garmendia, Peter Burkhard, Mazhar I. Khan

**Affiliations:** 1 Department of Pathobiology and Veterinary Science University of Connecticut, Storrs, CT, United States of America; 2 Department of Microbiology and Immunology, Faculty of Pharmacy, Al-Azhar University, Cairo, Egypt; 3 The Institute of Material Sciences, University of Connecticut, Storrs, CT, United States of America; 4 Charles River Laboratories, Avian vaccine services, North Franklin, CT, United States of America; 5 Department of Molecular Cell Biology, University of Connecticut, Storrs, CT, United States of America; 6 Alpha-O-Peptides AG, Riehen, Switzerland; Instituto Butantan, BRAZIL

## Abstract

Infectious bronchitis virus (IBV) affects poultry respiratory, renal and reproductive systems. Currently the efficacy of available live attenuated or killed vaccines against IBV has been challenged. We designed a novel IBV vaccine alternative using a highly innovative platform called Self-Assembling Protein Nanoparticle (SAPN). In this vaccine, B cell epitopes derived from the second heptad repeat (HR2) region of IBV spike proteins were repetitively presented in its native trimeric conformation. In addition, flagellin was co-displayed in the SAPN to achieve a self-adjuvanted effect. Three groups of chickens were immunized at four weeks of age with the vaccine prototype, *IBV-Flagellin-SAPN*, a negative-control construct *Flagellin-SAPN* or a buffer control. The immunized chickens were challenged with 5x10^4.7^ EID50 IBV M41 strain. High antibody responses were detected in chickens immunized with *IBV-Flagellin-SAPN*. In *ex vivo* proliferation tests, peripheral mononuclear cells (PBMCs) derived from *IBV-Flagellin-SAPN* immunized chickens had a significantly higher stimulation index than that of PBMCs from chickens receiving *Flagellin-SAPN*. Chickens immunized with *IBV-Flagellin-SAPN* had a significant reduction of tracheal virus shedding and lesser tracheal lesion scores than did negative control chickens. The data demonstrated that the *IBV-Flagellin-SAPN* holds promise as a vaccine for IBV.

## Introduction

Infectious bronchitis (IB) is a highly contagious avian disease that causes significant economic losses to the poultry industry. Commercial losses are mainly due to decreased weight gain and egg production, and a poor egg quality [1]. Infectious bronchitis virus (IBV), the causative agent of IB, belongs to the genus gamma coronavirus, and the order Nidovirales [2]. In addition to causing respiratory disease and reproductive system defects in chickens, some nephropathogenic IBV strains also affect the renal system and cause nephritis [3]. Infection with IBV is complicated when an opportunistic pathogen like *E*. *coli* is present [4].

Vaccination is utilized as a major means of controlling IB. Both live attenuated and inactivated virus vaccines are used to protect chickens against IB. However, limitations of live attenuated IBV vaccines include; reversion to virulence, tissue damage which can lead to secondary bacterial infections. In addition, the potential interference of maternal antibody on vaccine efficacy should also be taken into consideration [5]. Moreover, there is a possibility of recombination between virulent strains and vaccine strains, which may lead to the development of new pathogenic variants of IBV [6–8]. On the other hand, inactivated vaccines require priming with live attenuated vaccines, large doses of adjuvants, multiple vaccinations due to the shorter duration of the immune responses [9, 10]. These elevate the costs of vaccine production and limit its application [11]. Although DNA vaccines offer a novel method of immunization and can induce a cytotoxic T cell response, the low efficiency of these vaccines limit their application in the field [12]. Synthetic peptide vaccines alone are not immunogenic enough and require co-administration of adjuvants [13]. Therefore, there is demand for safe and more effective vaccines to control IB.

Lately, there has been an increasing interest directed towards the use of nanoparticles as vaccines against infectious pathogens [14–21]. These vaccines mimic various properties of viral pathogens, including size, and molecular configuration and the ability to induce both cellular and humoral immunity [22]. Our Self-Assembling Protein Nanoparticles (SAPNs) represent a novel vaccine platform composed of protein monomers that are capable of self-assembling into structures that mimic the size and shape of small viruses [14,16,18,19,21]. In recent years, we demonstrated the potential of SAPNs as a platform for vaccines, and successfully developed potent vaccine prototypes for Severe Acute Respiratory Syndrome [15], HIV [18], toxoplasmosis [19, 23], malaria[17,24] and influenza [16, 21]. SAPNs have many advantages in vaccine delivery over traditional methods. They can repetitively display epitopes on their surface when they are genetically fused to either the N- or C-terminus of the protein chain. Most importantly, the presentation is conformation-specific [14, 16, 21]. The sequence of antigens can be rapidly modified on response to the particular circulating pathogen and manufacturing can easily be scaled-up. Additionally, the incorporation of immunostimulatory molecule flagellin, a pathogen associated molecular pattern (PAMP), into the SAPN can generate a self-adjuvanted nanoparticle vaccine [21, 25].

In the present study, we developed a self-adjuvanted SAPN vaccine for the delivery of an antigenic polypeptide of IBV. IBV has a positive, non-segmented, single-stranded RNA genome 27 kb in length, coding for four structural proteins, the spike glycoprotein (S), the membrane protein (M), the envelope protein (E) and the nucleocapsid protein (N) [26]. The spike protein plays an essential role in attachment, virus-cell membrane fusion and host specificity during IBV infection. The IBV spike protein is a class I fusion protein which is characterized by formation of an α-helical coiled-coil structure [27]. It consists of the two subunits S1 and S2, which are in the outer membrane and anchored in the viral membrane. The S1 subunit is hypervariable whereas the S2 subunit is relatively conserved [28]. S1 induces neutralizing and serotype-specific antibodies, which lack cross-protective capacity amongst different strains [29] The S2 subunit contains two heptad repeat areas that contribute to the fusion process [30]. Because the S proteins on IBV are the most significant antigenic elements to induce neutralizing antibody, vaccine studies have focused on this protein [31–33].

In a previous study, we reported that a recombinant DNA vaccine carrying IBV S protein with chicken interferon protected chickens against challenge with Massachusetts 41 (M41) field type IBV [33]. Also, we have successfully designed a prototypic vaccine for SARS virus [15], which also belongs to the Coronaviridae family and displays a high degree of homology with the IBV spike glycoproteins. We have shown that the heptad repeat coiled coil (HRC) sequences of the SARS’ coronavirus S protein presented by SAPNs induces strong neutralizing antibodies against the SARS virus as tested in an *in vitro* infection inhibition assay [15] Based on sequence homologies between SARS coronavirus and IBV, in this study we investigated the potential of using self-adjuvanted SAPNs to present the HRC sequence of IBV’s S2 protein as a novel nanoparticle vaccine to control IBV.

## Materials and methods

### Ethics statement

Animal housing and all animal experiments were conducted strictly in accordance with Ag Guide (2010) and animal protocol approved by the Institutional Animal Care and Use Committee of University of Connecticut, Storrs, CT, USA (IACUC protocol #: A15-001). All animals were acclimated for one week prior to any experimental procedures. Chickens had ad libitum access to water and feed, and were monitored daily by attending veterinarians during the study. All animals were supervised daily by the attending veterinarians providing care to reduce pain and stress. All animals were humanely euthanized using CO_2_ in an inhalation chamber according to the approved protocol.

### Construct design and gene synthesis

The backbone of SAPN monomers were designed containing a pentameric and a trimeric coiled coil region held together by a linker and malaria (*Plasmodium falciparum*) epitope CelTOS (residues 24–83 and 84–182), amino acid residues were showed in S1 Fig. One monomer (Monomer A, S1 Fig) was designed based on the antigen sequence (residues 1056–1095) corresponding to the C-terminus of the S protein of the IBV-M2118 strain which was named IBV-SAPN monomer. The second monomer contained the D0 and D1 domains (residues 4–186 and 192–282) of flagellin from *Salmonella enterica* serovar Typhimurium in place of the IBV antigen (Monomer B, S1 Fig) named flagellin-SAPN monomer. To render the nanoparticle more immunogenic, the two CD4+ epitopes AKFVAAWTLKAAA and HAAHAAHAAHAAHAA were engineered into the scaffold of the SAPN. The sequences coding for both constructs were codon optimized, for expression in *E*. *coli*, synthesized and expressed by GenScript USA (Piscataway, NJ).

### Protein expression and purification

Expression and purification was carried out as previously described [21]. Briefly, protein was expressed in BL21 (DE3) pLys in a 1 L LB culture supplemented with ampicillin (200 μg/mL) and chloramphenicol (30 μg/mL). Expression was induced by adding 1 mM isopropyl β-D-thiogalactopyranoside when culture reached an OD600 of 0.8. After 4 h of incubation, cultures were pelleted. Pellets were re-suspended in lysis buffer 8M urea, 100 mM NaH_2_PO_4_, 20mM Tris base, pH 8.0, and then were lysed by sonication. Lysate was cleared by centrifugation at 30,500xg for 25 min at 4°C. Clear lysate was purified in a 5 mL HisTrap HP column (GE Healthcare, Piscataway, NJ) using a purifier, ÄKTApurifier 100 (GE Healthcare, Piscataway, NJ). Five column volumes of binding buffer of 8M urea, 100 mM NaH2PO4, 20 mM imidazole, 20mM Tris base, pH 8.0 were used to equilibrate HisTrap HP column. Clear lysate was loaded into the equilibrated column and then underwent washing with 5 volumes of binding buffer. Columns were then washed with 5 column volumes of pH gradient phosphate buffer pH 8.0 buffer (8M urea, 500 mM NaH_2_PO_4_, 20mM Tris base), pH 6.5 buffer (8M urea, 100mM NaH_2_PO_4_, 20 mM sodium citrate), pH 5.5 buffer (8M urea, 100mM NaH_2_PO_4_, 20mM sodium citrate, pH 5.5), and pH 4.5 buffer (8M urea, 100mM NaH_2_PO_4_, sodium citrate, pH 4.5), and finally a buffer 8M urea, 100 mM NaH2PO4, 20mM Tris base, pH 8.0. Subsequently, a 500 mM imidazole gradient was used to elute protein from the column. Fractions of the eluate were analyzed by SDS-PAGE. Fractions containing the target proteins were combined and dialyzed overnight to remove imidazole in a pre-refolding buffer (8 M Urea, 50 mM NaCl, 20 mM Tris, 5% Glycerol pH 8.0).

### Co-assembly and SAPN refolding

The protein concentration was determined by NanoDrop 2000 (Thermo Scientific, Wilmington, DE). To generate Self-Adjuvanted SAPNs (*IBV-Flagellin-SAPN*) a molar ratio of 58:2 IBV-SAPN monomer to Flagellin-SAPN monomer, was established based on protein concentration. After establishment of the molar ratios, SAPNs were refolded in a stepwise manner by slowly removing urea, in which the gradient urea concentration of refolding buffers were used 8M, 6M, 4M, 2M, 120 mM Urea in 20 mM Tris, 5% glycerol pH 8.0. SANPs were dialyzed for 4 hours in each concentration of urea.

### Analysis of SAPN by dynamic light scattering (DLS) and Transmission Electron Microscopy (TEM)

SAPNs were analyzed as previously described [21]. The SAPN’s hydrodynamic diameter was measured by DLS at 25° C, and presented as the average of five runs. SAPNs were absorbed on a carbon coated grid, stained with 0.5% uranyl acetate, and imaged using a FEI Tecnai 12 G2 Spirit BioTWIN.

### Immunization and challenge

Four-week-old SPF white Leghorn chickens (n = 5) (Charles River laboratories, CT, USA) were given 200μL vaccine formulation containing 100 μg *IBV-Flagellin-SAPN* intramuscular injection. Each animal received three doses at nine day intervals (at days 1, 11 and 21 of the experiment). Chickens were bled once before vaccination (at days one of the experiment) and bled three times nine days after each immunization (at days 10, 20 and 30 of the experiment). The sera from the experimental chicken were stored at -80° C until tested. As negative controls, *Flagellin-SAPN*, or refolding buffer was used to immunize chicken (5 chickens for each group) with the same immunization protocol. Another non-vaccinated/non-challenged negative control group was included in the study for histopathology comparison. Chickens were challenged at intranasally with 5x10^4.7^ EID_50_ IBV M41 strain per chicken at 58 days of age. The chickens were observed daily for clinical signs, tracheal swab samples were collected at two and four days post challenge and the chicken were euthanized four days post-challenge. Tracheal swabs were placed in brain heart infusion broth media, and stored at -80 °C until tested. Chicken groups were housed separately in isolators with controlled pressure, temperature and humidity in accordance with Ag Guide (2010). Any chicken showing lacking of interest in food and water, difficulty in breathing, or depression were brought to the attention of the veterinary staff at animal care office.

### Lymphocyte proliferation

Peripheral blood mononuclear cells (PBMC) were isolated using gradient centrifugation in Histopaque-1077 (Sigma). Briefly, 1 mL of blood was mixed with equal amount of 1X PBS. The blood-PBS mixture was overlaid on top of an equal volume of Histopaque-1077 and centrifuged for 30 min at 400 ×g at 20° C. The opaque interface was collected and washed twice with PBS with centrifugation at 300 xg for 5 min for each wash. The cell pellet was re-suspended, stained with trypan blue and counted with a hemocytometer. PBMCs (2x10^5^ cells/well) were plated in 96-well cell culture plates. The PBMCs were stimulated in triplicate wells with 10 μg/mL concanavalin A, 10 μg/mL protein nanoparticle constructs, or 10 μg/mL UV-inactivated IBV M41 strain, respectively. Plates were incubated at 41° C with 5% CO_2_ for 48 hours. The proliferation assay was conducted using a cell proliferation ELISA BrdU (colorimetric) kit (Roche Applied Science, Mannheim, Germany). Briefly, BrdU was added into each well with a final concentration of 10 μM and incubated at 41° C with 5% CO_2_ for 21h. Plates were centrifuged at 300g for 10 min. Labeling medium was removed by flicking- off plates, which were air-dried in the dark overnight. 200 μL/well FixDenat was used to fix cells in the plates for 60 min at 15–25° C. The fixative was removed thoroughly by flicking and tapping. One-hundred μL/well anti-BrdU-POD working solution was added to the microplates and incubated for 90 min at 15–25 °C. Plates were washed three times using PBS. A substrate solution was added (100 μL/well TMB (tetramethyle-benzidine) solution) and incubated at 15–25° C for 5–30 min until color development was visible. The reaction was stopped by adding 25 μL of 1M sulfuric acid to each well. Absorbance was measured at 450nm with background reference wavelength 690nm. Stimulation index (SI) was calculated by the formula, SI = OD value (antigen-stimulated PBMCs)/OD value (medium treated PBMCs).

### Enzyme-linked immunosorbent assay (ELISA)

ELISA plates (Thermo Scientific, USA) were coated with 50 μL containing 10 μg/mL synthetic HR2 sequence (FVRALSMQILDIDSEIDRIQGVIQGLNDSLIDLEKLSILKTYIKWPWY) in carbonate-bicarbonate buffer pH 9.6 overnight at 4°C. Plates were blocked with 100 μL of a 3% bovine serum albumin (BSA) in PBST solution (PBS plus 0.05% Tween 20). Chicken’s sera collected during the vaccine trial were serially diluted starting at 1/40 and were incubated in the plates at room temperature for 1 hour. Hyperimmune sera were used as positive control was included in each ELISA test. After washing the plates with PBST, secondary antibody (mouse anti-chicken Ig-Y, Southern Biotech, AL, USA) was added and incubated in the plates at a 1/1000 dilution at room temperature for 1 hour. The plates were washed and then 50 μL ELISA TMB (Thermo Scientific) substrate was applied to the wells at room temperature for 5 to 15 minutes until color developed. Reactions were stopped with 0.01M HCl. Plates were read for absorbance at 450 nm.

### Virus recovery

Three nine day-old SPF embryonated chicken eggs were inoculated in the allantoic cavity with 200μl of each filter sterilized tracheal swab. Eggs were incubated at 37°C for five days. The eggs were observed daily for embryo mortality. All eggs were kept at 4°C on the fifth day post-inoculation. The allantoic fluids were harvested.

### Quantification of viral copies by real time RT-PCR

RNAs were extracted from the tracheal swabs and from recovered allantoic fluids using QIAamp viral RNA mini kit (Qiagen, Hilden, Germany). First strand cDNA was synthesized in a reverse transcription reaction (RT) using the high capacity cDNA reverse transcriptase kit of Applied Biosystems (USA). Total RNA 680 ng was used in a 20 μL reaction containing 0.8 μL dNTP, 2 μL reaction buffer, 2 μL random primer and 1 μL enzyme mix according to the manufacturer’s instructions. The RT reaction was conducted in a thermocycler (Applied Biosystems) with a programmed profile of 26° C for 10 min, 42° C for 45 min, and 75° C for 10 min. Real time RT-PCR was performed as previously described [34]. The reaction mix was prepared with 10 μL master mix (SYBR green PCR master mix, Applied Biosystems), 0.25 μL of 25 pmole forward and reverse primers (Forward: ACA GGT TCT GGT GGT GTT TAG TG; Reverse: AGT TGT TCG GGA ATG TCT TTG G), 4 μL of cDNA template and nuclease-free water was added to bring the volume to 20 μL. Reaction was run using a Bio-Rad real time PCR system CFX 96 with a cycling profile, at 94° C for 2 min, and 40 cycles at 94° C for 20 s, 60° C for 20 s, and 72° C for 15 s. Melting temperature was determined from 65° C to 95° C with heating ramp of 0.5° C/s.

### Histopathology

Tracheal tissues were harvested after euthanasia and necropsy four days post challenge, and fixed in 10% neutral formalin. Hematoxylin and eosin staining was performed on cross sections of tracheal tissues. A scoring scale was established with modification of previously described scoring method [35].

### Statistical analysis

Data was analyzed using one or two-way ANOVA with Bonferroni post-test in GraphPad prism 6.0. Statistical significance was achieved at levels of P values <0.05 (*), <0.01 (**), and <0.001 (***).

## Results

### Synthesis and biophysical characterization of nanoparticles

**Fig 1:**
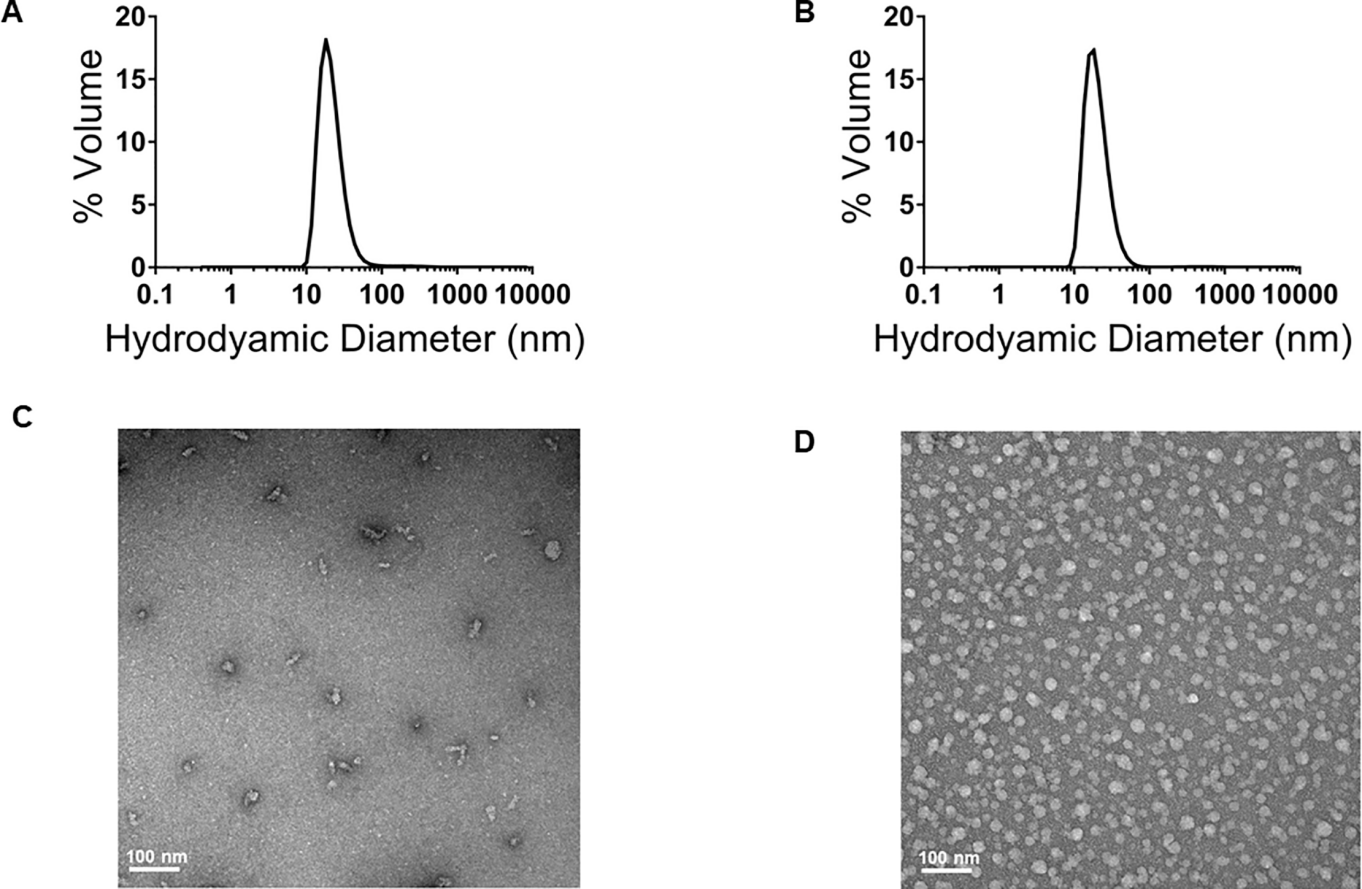
Characterizations of *IBV-Flagellin-SAPN* and *Flagellin-SAPN* constructs. (A) Dynamic light scattering of *IBV-Flagellin-SAPN*. (B) Dynamic light scattering of *Flagellin-SAPN*. (C) Transmission electron microscopy of *IBV-Flagellin-SAPN* and (D) Transmission electron microscopy of *Flagellin-SAPN*.

The genes of IBV-SAPN monomer and Flagellin-SAPN monomer were successfully synthesized and sub-cloned into plasmids (S1 Fig). SDS-PAGE of refolded SAPNs revealed that *IBV-Flagellin-SAPN* and *Flagellin-SAPN* showed high purity and expected molecular weights of 37.8 kDa and 64.5 kDa for the two types of protein chains (S2 Fig). Dynamic light scattering showed a hydrodynamic diameter of 22.8 nm and 20 nm of *IBV-Flagellin-SAPN* and *Flagellin-SAPN* respectively, which was confirmed by transmission electron microscopy (Fig 1).

### Vaccination of chickens with *IBV-Flagellin-SAPN* elicited high levels of antibody

**Fig 2:**
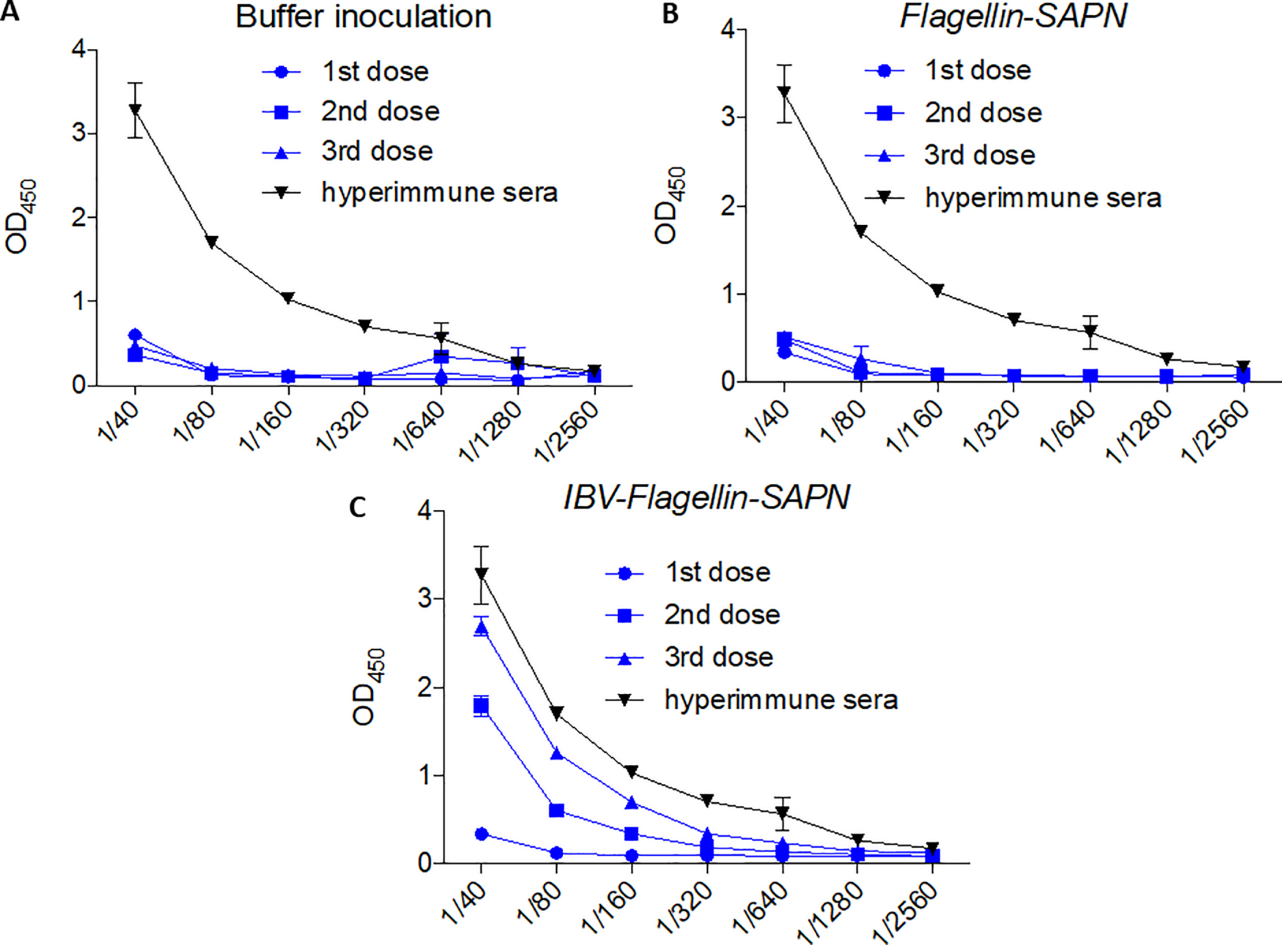
Antibody responses in immunized chickens by ELISA. (A) Buffer inoculated group. (B) *Flagellin-SAPN* inoculated group. (C) *IBV-Flagellin-SAPN* immunized group.

To test for antibody responses elicited by the SAPNs, ELISA was performed using 96-well plates coated with 10 μg/mL of synthetic HR2 peptide. The results show markedly elevated antibody responses in chickens vaccinated with *IBV-Flagellin-SAPN* (Fig 2C) while *Flagellin-SAPN* and buffer control chicken groups had no detectable antibody response (Fig 2A and 2B). Booster effects were observed in chickens prime-vaccinated with *IBV-Flagellin-SAPN* as indicated by elevations of antibody level after each additional dosage (Fig 2C). These results demonstrated that *IBV-Flagellin-SAPN* vaccine induced humoral immune responses in chickens.

### Ex vivo recall responses of PBMCs from chickens vaccinated with *IBV-Flagellin-SAPN* were significant

**Fig 3:**
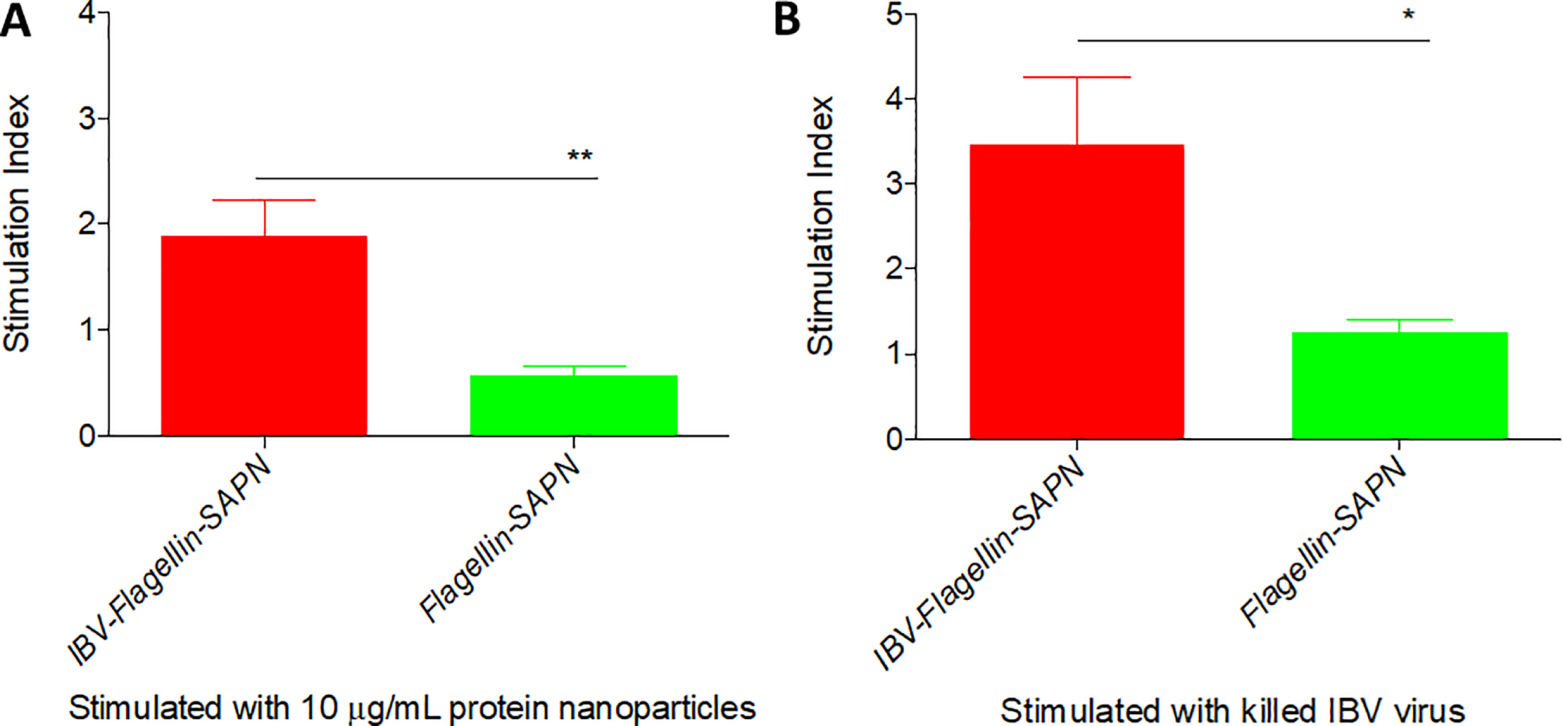
Lymphocyte proliferation of immunized chickens. (A) PBMCs were pulsed with the respective SAPN at a final concentration of 10 μg/mL. (B) PBMCs were pulsed with killed IBV virus. Stimulation index was calculated by the formula = OD value (antigen-stimulated PBMCs)/OD value (medium treated PBMCs).

The stimulation of PBMCs from chickens immunized with *IBV-Flagellin-SAPN* with SAPNs (Fig 3A) or inactivated IBV virus (Fig 3B) resulted in a significantly higher proliferation than that of the corresponding PBMC from the negative control group (*Flagellin-SAPN)*. This demonstrated the induction of cellular immune responses and generation of a memory pool by the vaccine.

### A reduction of shedding of IBV was detected in trachea of chicken vaccinated with *IBV-Flagellin-SAPN*

**Fig 4:**
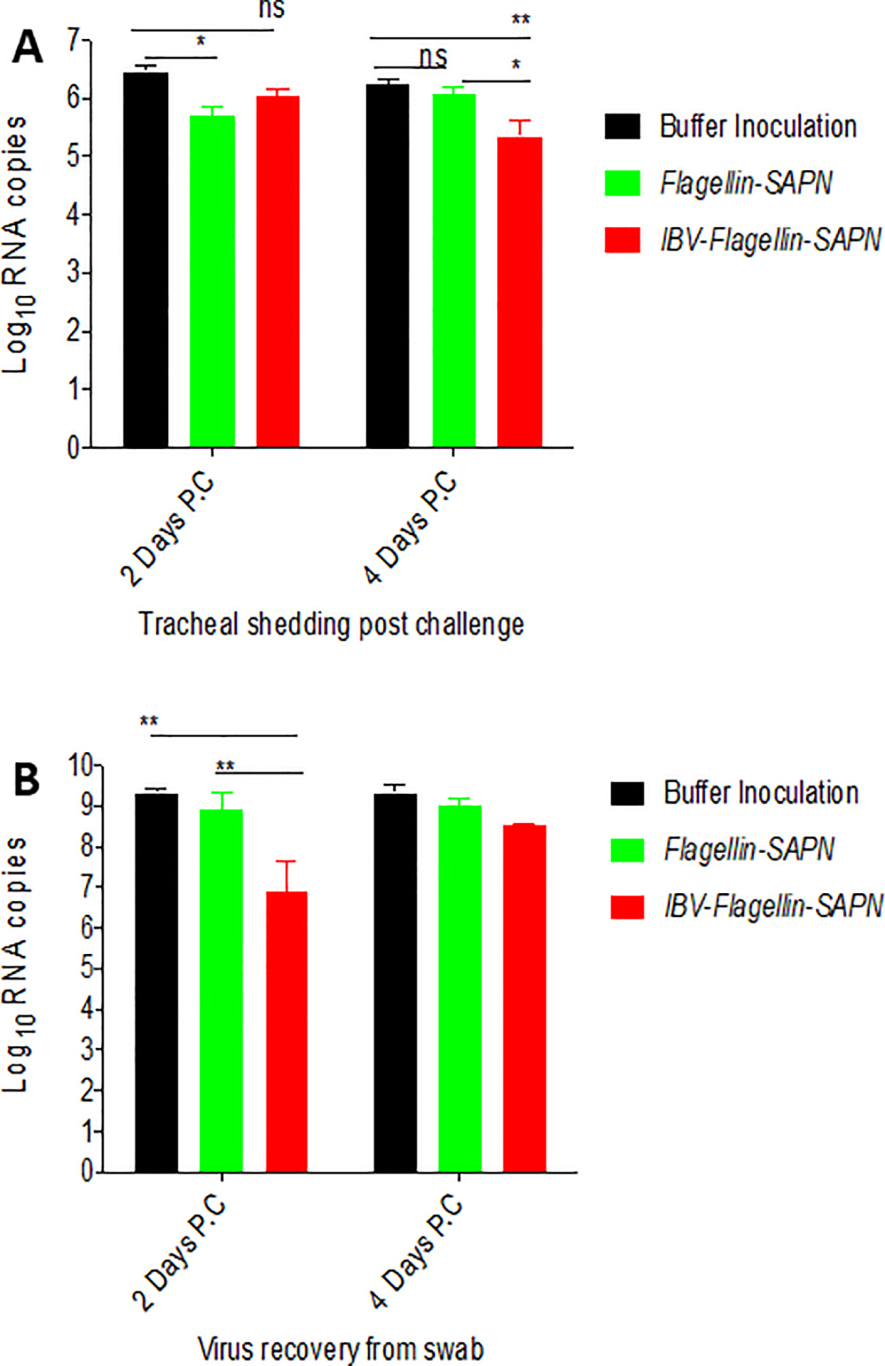
Viral RNA copy numbers in chicken tracheas and virus recovery from tracheal swabs in chicken embryos. (A) Tracheal swab samples were used to extract RNA that was quantified by real time RT-PCR. (B) Virus was recovered and isolated by inoculating tracheal swab samples into embryonated eggs. Recovered virus was quantified by real time RT-PCR.

To evaluate the vaccine effects on restricting virus replication, viral copy numbers were measured by qRT-PCR in tracheas of immunized or negative control chickens after challenge with IBV M41. A significant reduction of virus shedding in tracheas was found at four days post challenge with *IBV-Flagllin-SAPN* as compared to *Flagellin-SAPN* and Buffer groups (Fig 4A). Interestingly, chickens inoculated with a negative control construct, *Flagellin-SAPN*, also had lower level of virus shedding than the buffer inoculated group. A significant reduction of virus induced by *Flagellin-SAPN* was found at two days post challenge (Fig 4A). To rule out the inference of non-infectious virus on the result, we also inoculated embryonated eggs with tracheal swab material, and quantified the RNA copy number of the recovered virus by real time RT-PCR. Interestingly, we found that the RNA copy numbers recovered from both groups receiving SAPN (*IBV-Flagellin-SAPN* and *Flagellin-SAPN*) was lower than that of the buffer control group, indicating a level of restriction of virus replication in chickens by SAPN treatment itself (Fig 4B). A significantly lower viral load was observed in *IBV-Flagellin-SAPN* group at two days but not at four days after inoculation of the embryonated eggs compared to buffer control or *Flagellin-SAPN* groups (Fig 4B).

### Chickens vaccinated with *IBV-Flagellin-SAPN* had significantly lesser lesion scores in tracheal tissue than control groups

**Fig 5:**
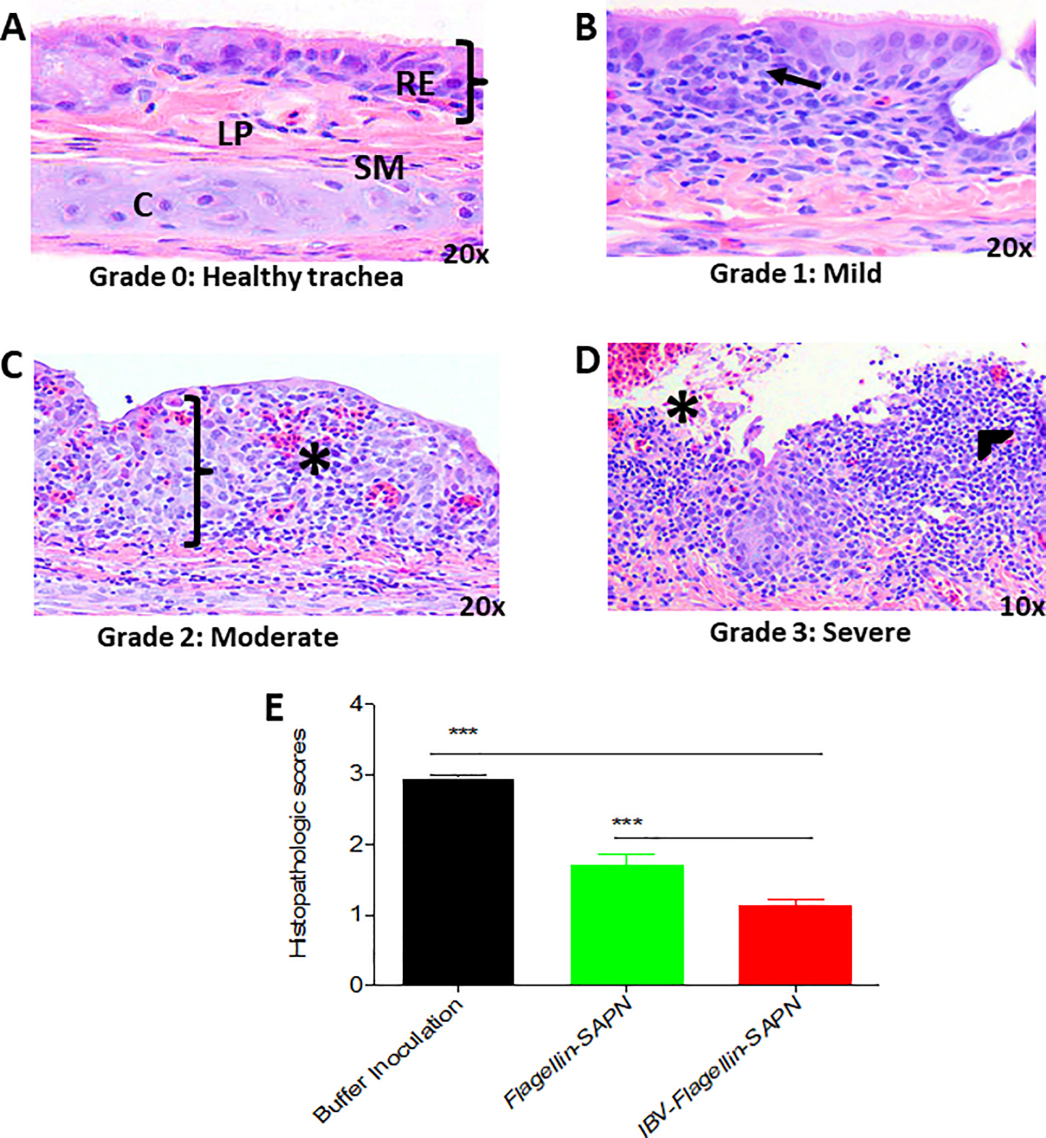
Evaluation of histopathologic lesion in tracheal tissues. (A) Normal tracheal tissue from chickens showed no lesions (coming from Negative group: non-vaccinated/ non-challenged group); score 0. (B) Tracheal tissues from chickens showing infiltration of inflammatory cells (black arrow) such as lymphocytes, macrophages and few heterophils transmigrating and infiltrating lamina propria and submucosa developing germinal centers; score 1-mild. (C) Tracheal tissues from chickens showing desquamation of ciliated cells, marked epithelial hyperplasia, infiltration of lymphocytes, macrophages and heterophils (mucosa and submucosa), congestion and hemorrhage (black line); score 2-moderate. (D) Tracheal tissue from chickens’ showing large number of infiltrates (mucosa and submucosa), mucosa completely obliterated, fibrous exudate in lumen admixed with RBC and necrotic debris, congestion and hemorrhage (black star), score 3-severe. (E) Histopathologic score. RE: respiratory epithelium (black braces); LP: lamina propria; SM: submucosa and C: cartilage.

The restriction of progression of disease is a direct indicator of the prophylactic effect of a tested vaccine candidate. To evaluate the vaccine-induced protection, we scored microscopic lesions in tracheal tissues at 4 days post challenge with four grading scales (Fig 5A–5D). We observed that, the histology of tracheal tissue of chickens vaccinated with the *IBV-Flagellin-SAPN* group was quite similar to the tracheal tissue histology of the non-vaccinated/non-challenged group. The *IBV-Flagellin-SAPN* group had significantly lesser tracheal lesion scores than the *Flagellin-SAPN* group or the buffer control group (Fig 5E). These results were consistent with the virus shedding in the trachea recorded at four days after challenge, suggesting the protective efficacy of our *IBV-Flagellin-SAPN* vaccine.

## Discussion

Vaccination has been largely used as a measure to control IBV for over 50 years. However, a challenge remains because currently available live attenuated or killed vaccines are not effective to combat rapidly emerging variant strains generated from point mutation or recombination events. In addition, live attenuated vaccines are still the predominant vaccines in use, and their use raises the concern of safety [36, 37] as they contribute to the emergence of new pathogenic IBV variants [6]. In a study of live vaccine efficacy, about 10% of the chickens did not show any immune response when exposed to IBV even when raised in the same environmental conditions [38]. These differences are dependent on chicken genetics and are highly variable [39]. Killed vaccines are another currently commercialized option for IBV protection. These vaccines do not appear to elicit much protection for the respiratory tract as less than 59% of chickens in a study displayed tracheal immunity [1]. Peptide subunit vaccines may be safer, but are usually poorly immunogenic when used alone [13]. Nanoparticle technologies have been demonstrated as a promising delivery system to improve drug efficacy. Nevertheless, there are still some issues challenging the development of nanoparticles as vaccine delivery systems. On one hand, cytotoxicity of some nanoparticles like titanium oxide, gold, or cationic lipids [40–43] is a major problem. On the other hand, maintaining antigen stability and correctly displaying antigens in their native conformation on the surfaces of some nanoparticles may be challenging [44].

In contrast, our SAPNs have been demonstrated as an innovative method in vaccine design. They repetitively display antigenic epitopes in their native conformation and have successfully been applied to construct vaccines that induce robust immune responses against numerous pathogens including malaria, influenza, SARS, toxoplasma and HIV [14, 16, 18, 19, 21, 23, 25].

In the present study, we have developed a SAPN based IBV vaccine candidate that repetitively presents IBV antigens. In this vaccine, the malaria epitope CelTOS was included in the in the design because it renders the SAPNs biophysically well-behaved. Based on our previous experience, CelTOS-containing SAPNs are nicely soluble and form spherical particles. Therefore, this otherwise unrelated malaria protein included into the design of the IB vaccines is beneficial. This vaccine also contains the bioactive domains of flagellin to self-adjuvant the vaccine and renders it more immunogenic. We successfully applied this system for influenza, malaria and toxoplasmosis, achieving a significant improvement on the immunogenicity as tested in terms of antibody response against these diseases [16, 21, 23, 25]. In the current study, we expanded our self-adjuvanted SAPN technology for use in avian infectious bronchitis.

Spike protein subunits of different viruses have been used in vaccination studies with success. Newcastle disease virus, for instance, has been modified to express the S subunit and was found to provide protection comparable to commercial vaccines after two vaccinations ([45]. Similar observations have been found using baculovirus [46]and fowl adenovirus [47]vectors. The IBV coronavirus S protein is considered the main virus attachment protein containing critical neutralizing epitopes, thus making it ideal as a vaccine component to induce protection against coronaviruses [29, 48, 49]. Indeed, several studies suggested that the S protein could serve as major target for vaccine design against coronaviruses [33, 50–54]. The S2 protein subunit, which is expressed on the surface of IBV, is a promising immunogen and can provide increased cross-protection against various serotypes [55, 56] since it is a highly conserved region [57]. The HR region of coronavirus S2 protein is an α-helical trimeric coiled-coil [58]. Therefore, a vaccine that displays HR in its native trimeric coiled-coil conformation would perform well in chicken. Our previous research showed that a SAPN that repetitively displays a SARS coronavirus B-cell epitope from the C-terminal HR of the virus’ spike protein was able to neutralize SARS-coronavirus infectivity [15]. Based on homologies between the SARS-coronavirus and IBV S proteins we hypothesized that the equivalent region of IBV S2 would have similar vaccine potential. In the present study, SAPNs that repetitively display HR2 of IBV S2 protein induced noticeably high level of antibody responses. This initial result demonstrated that the prototype of our *IBV-Flagellin-SAPN* nanoparticle vaccine was able to induce strong humoral immune responses in vaccinated chickens. Repetitive display of B cells epitopes is essential to enhance humoral immune responses [2, 59]. The results of our study support that appropriate HR2 epitopes were repetitively displayed likely in their native trimeric conformation by the SAPN vaccines as evidenced by the relatively strong antibody response elicited in chickens. Furthermore, our *IBV-Flagellin-SAPN* vaccine significantly stimulated cellular immunity and generated a memory pool as demonstrated by the response of PBMCs from vaccinated chickens to recall antigen stimulation.

In the current study, protection was higher in the group of chickens vaccinated with *IBV-Flagellin-SAPN* than that in control groups (*Flagellin- SAPN* and buffer groups). Also the viral RNA loads in tracheas were significantly lower as compared to control groups four days after challenge. This was consistent with significantly lesser tracheal lesion scores four days after challenge in chickens vaccinated with *IBV-Flagellin-SAPN* than *Flagellin- SAPN* and buffer groups. Microscopic evaluation revealed that, there were no major pathologic changes in the tracheas of *IBV-Flagellin SAPN* vaccinated chicken and the histology was comparable to that non-vaccinated/non-challenged control group. This is a clear indication of vaccine induced protection. Our results are consistent to those reported previously in chickens vaccinated with a recombinant S-ectodomain protein vaccine which elicited protection against IBV challenge and suggested that, S2 domain has a crucial role in persuading protective immunity (Eldemery et al., 2017)

On the basis of the viral RNA load in the trachea and tracheal histopathological assessments, the *IBV-Flagellin-SAPN* vaccine has shown here a clear potential to protect chickens against challenge with IBV M41 without co-administration of commercial adjuvants. However, it has to be kept in mind that this is an experimental vaccine and no previous data exist with the use of the same platform with this particular IBV antigen as to make comparisons. Therefore, this work conducted here provides proof of principle where vaccine immunogenicity was demonstrated as shown by measurable antibody and cellular responses and a level of protection were clearly demonstrated in vaccinated chickens.

In the current study we incorporated flagellin to the SAPN as an immunostimulatory molecule to generate a self-adjuvanted SAPN. Flagellin has been extensively explored for the use as an adjuvant in various kinds of vaccine formulations, including soluble mixture with proteins, co-expressed with epitopes or encoded in DNA vaccines [60–62]. It is interesting that the *Flagellin-SAPN* is able to significantly reduce viral shedding in trachea of inoculated chickens after challenge with IBV (Fig 4). Chickens inoculated with *Flagellin-SAPN* also had significantly lesser lesion scores in tracheal tissues than chickens inoculated with buffer (Fig 5E). The protective effect of *Flagellin-SAPN* may be attributed to the non-specific activation of natural killer cells and macrophages, and to the recruitment of heterophiles, which lead to the limited and restricted virus replication. These observations are in accordance with the fact that flagellin can induce pro-inflammatory cytokines accounting for an antiviral effect. These data support an intrinsic quality of the vaccine backbone which is a significant asset. The level of protection with the *IBV-Flagellin-SAPN* vaccine was still significantly higher than that of *Flagellin-SAPN* based on both parameters. These data suggested that we have successfully implemented a self-adjuvanted SAPN for the use as a vaccine backbone against IBV.

Although, our nanoparticle vaccine structure was effective in delivering the S2 subunit peptides, other peptides from the S2 or a mixture of S1 and S2 subunit components on the same nanoparticle may be investigated for improved vaccine performance immunogenicity of our effective backbone.

The SAPN is such an effective and versatile backbone that will enable us to explore additional viral antigens to further enhance the vaccine efficacy and eventually apply it to control the disease.

## Supporting information

S1 Fig Amino acid sequence of each SAPN monomer. (A) IBV-SAPN monomer (Monomer A). (B) *Flagellin-SAPN* monomer (Monomer B); Color scheme: Red, malaria protein; Green: a pentameric coiled coil; yellow: trimeric coiled coil; Blue: HR2 B cell epitope; Black: linker sequences; Pink: CD4 T cell epitopes; Purple: Sequence of the flagellin D0 and D1 domains.
(TIF)

S2 Fig SDS-PAGE analysis of assembled SAPNs. 1- IBV-SAPN monomer (Monomer A, Mw 37.8 kDa); 2- *IBV-Flagellin-SAPN* assembled from Monomer A and Monomer B at a 58:2 molar ratio; and 3- Flagellin-SAPN monomer (Monomer B, Mw 64.5 kDa.
(TIF)
