# Supplementary figures and images for: A self-adjuvanted nanoparticle based vaccine against infectious bronchitis virus
Source: PLoS One. 2018 Sep 14;13(9):e0203771. doi: 10.1371/journal.pone.0203771 (PMC6138407; doi:10.1371/journal.pone.0203771)

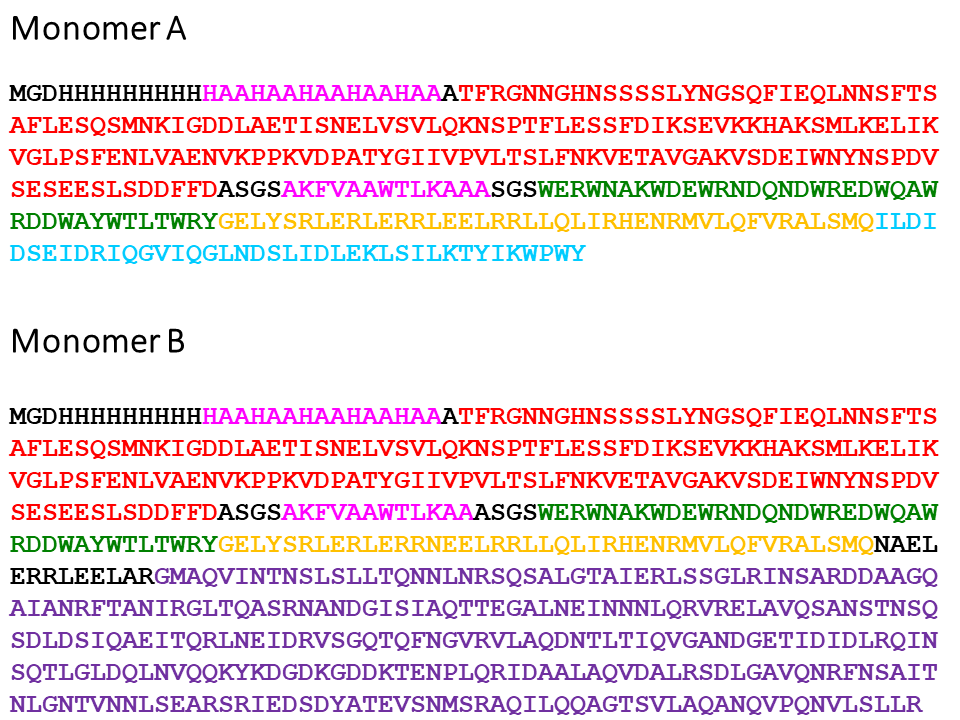

Supplement: S1 Fig — (A) IBV-SAPN monomer (Monomer A). (B) Flagellin-SAPN monomer (Monomer B); Color scheme: Red, malaria protein; Green: a pentameric coiled coil; yellow: trimeric coiled coil; Blue: HR2 B cell epitope; Black: linker sequences; Pink: CD4 T cell epitopes; Purple: Sequence of the flagellin D0 and D1 domains. (TIF) [file pone.0203771.s001.tif]

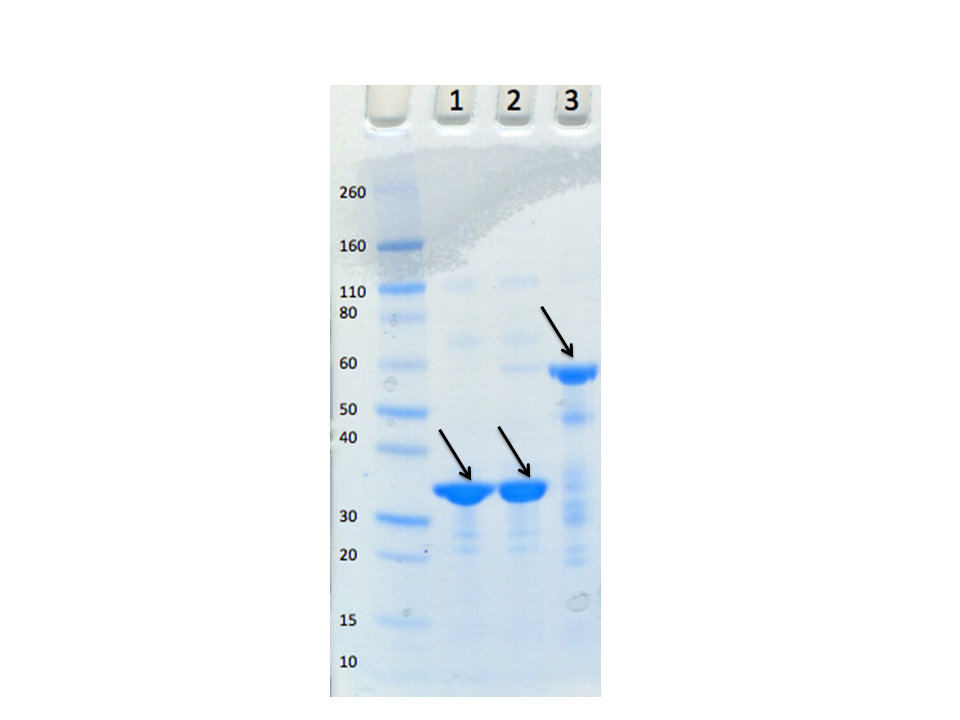

Supplement: S2 Fig — 1- IBV-SAPN monomer (Monomer A, Mw 37.8 kDa); 2- IBV-Flagellin-SAPN assembled from Monomer A and Monomer B at a 58:2 molar ratio; and 3- Flagellin-SAPN monomer (Monomer B, Mw 64.5 kDa. (TIF) [file pone.0203771.s002.tif]
